# Optimizing internet-delivered cognitive behaviour therapy for alcohol misuse—a randomized factorial trial examining effects of a pre-treatment assessment interview and guidance

**DOI:** 10.1186/s13722-022-00319-0

**Published:** 2022-07-23

**Authors:** Christopher Sundström, Vanessa Peynenburg, Carly Chadwick, David Thiessen, Andrew Wilhems, Marcie Nugent, Matthew T. Keough, Michael P. Schaub, Heather D. Hadjistavropoulos

**Affiliations:** 1 Department of Clinical Neuroscience, Karolinska Institutet, Centre for Psychiatric Research, Norra Stationsgatan 69, 113 64 Stockholm, Sweden; 2 Department of Psychology, Stockholm University, 106 91, Stockholm, Sweden; 3 Department of Psychology, University of Regina, 3737 Wascana Parkway, Regina, SK S4S 0A2 Canada; 4 Department of Psychology, York University, 4700 Keele St, Toronto, ON M3J 1P3 Canada; 5 Swiss Research Institute for Public Health and Addiction, University of Zurich, Konradstrasse 32, 8005 Zurich, Switzerland

**Keywords:** Alcohol, Treatment, Internet, Cognitive behavior therapy, Guidance, Assessment reactivity

## Abstract

**Background:**

Internet-delivered cognitive behavioral therapy (ICBT) for alcohol misuse has potential to radically improve access to evidence-based care, and there is a need to investigate ways to optimize its delivery in clinical settings. Guidance from a clinician has previously been shown to improve drinking outcomes in ICBT, and some studies suggest that pre-treatment assessments may contribute in initiating early change. The objective of this study was to investigate the added and combined effects of a pre-treatment assessment interview and guidance on the outcomes of ICBT for alcohol misuse delivered in an online therapy clinic.

**Methods:**

A 2X2 factorial randomized controlled trial was conducted where participants received access to an 8-week ICBT program, and either a pre-treatment assessment interview (Factor 1), guidance (Factor 2), a combination of these, or neither of these. Participants were 270 individuals (66.8% female, mean age = 46.5) scoring 8 or more on the Alcohol Use Disorders Identification Test and consuming 14 standard drinks or more in the preceding week. Primary outcomes were number of drinks consumed and number of heavy drinking days during the preceding week, 3 months post-treatment.

**Results:**

Large within-group effects were found in terms of alcohol reductions *(d*_*w*_ ≥ 0.82, *p* < 0.001), but neither of the factors significantly improved drinking outcomes. Guidance was associated with greater adherence (i.e. completed modules).

**Conclusions:**

Neither a pre-treatment assessment interview nor guidance from a clinician appears to improve drinking outcomes resulting from internet-delivered cognitive behaviour therapy for alcohol misuse when delivered in a routine online therapy clinic.

*Trial registration:* NCT03984786. Registered 13 June 2019, https://clinicaltrials.gov/ct2/show/NCT03984786

**Supplementary Information:**

The online version contains supplementary material available at 10.1186/s13722-022-00319-0.

## Background

Alcohol misuse contributes greatly to the global burden of disease [1, 2], but only one in six receive treatment [3]. Internet interventions could radically increase access to evidence-based treatment for the alcohol misuse population, and with a recent individual patient-data meta-analysis (IPDMA) demonstrating that they render significant alcohol reductions [4], the question no longer appears to be whether these interventions are effective, but rather how their effects can be optimized when implemented in routine online therapy clinics [5] and other clinical settings.

The most common way of optimizing effects in internet interventions is through adding guidance from a clinician [6]. In the previously mentioned IPDMA, guided interventions were found superior to unguided ones (comparative reduction: − 6.78 drinks) [4]. However, there are several reasons that more research on guidance is needed: first, the IPDMA did not distinguish between the two main intervention formats: internet-based cognitive behavioral therapy (ICBT) and personalized normative feedback (PNF). In fact, only three studies in the IPDMA compared guided to unguided ICBT; two found small and medium differential effects in favor of guidance [7, 8], and one did not find differential effects [9]. Second, several recent trials have failed to find differential effects between guided and unguided ICBT [10–12], indicating that effects of guidance may not be as robust as suggested by the IPDMA. Further, none of the above mentioned studies were conducted in routine care clinics, suggesting a need to investigate the effects of guidance when ICBT is delivered in clinical settings.

Exposure to structured assessments represents another factor that may improve drinking outcomes in alcohol treatment, a phenomenon referred to as assessment reactivity (AR) [13–15]. Although the exact mechanisms of AR are unknown, it has been hypothesized that being asked to reflect on one’s own drinking may lead to recognition of a discrepancy between current behaviour and personal values, which, in turn initiates change [16]. AR has been observed at pre-treatment, post-treatment and follow-up assessments [17], and research suggests that semi-structured pre-treatment assessment interviews render immediate reductions in drinking. For example, in one study, where females with alcohol problems received three assessment interviews prior to being randomized to receive either individual or couples CBT, 44% of participants became abstinent prior to treatment start, and these participants also had significantly better drinking outcomes both during treatment and at 12-month follow-up [18]. In a trial of adolescents with substance use problems where all participants underwent an intake assessment interview before treatment start, 51% had become abstinent by the first session, with further analyses showing that those abstinent by the first session had significantly better drinking outcomes at the last session of the treatment than those not abstinent [19]. In a third study, significant reductions were observed in a non-help seeking control group after participating in an extensive intake interview [20]. Although none of these studies employed an experimental design, the findings appear consistent with an AR effect. Furthermore, the initial alcohol reductions observed were related to longer-term reductions in two of the studies [18, 19], suggesting that pre-treatment assessment interviews have potential as a treatment component that could help optimize treatment effects of internet interventions. However, there are no studies investigating the impact of pre-treatment assessment interviews in ICBT for people with alcohol misuse.

The aim of the current factorial randomized controlled trial was to examine optimal delivery of ICBT for alcohol misuse in a routine online therapy clinic through the application of two specific factors: an assessment interview guided by the alcohol use disorder module of the Structured Clinical Interview DSM-5 (SCID-5) (Factor 1) and guidance from a clinician (Factor 2).

We hypothesized that at the 3-month follow-up:

- participants receiving the assessment interview would have reduced their drinking more than those not receiving the assessment interview;
- participants receiving guidance would have reduced their drinking more than those not receiving such guidance; and
- participants receiving a combination of the two factors (assessment interview and guidance), would have reduced their drinking more than any of the other three treatment conditions.

Secondarily, we evaluated immediate effects of the assessment interview on drinking and motivation to change, hypothesizing that, at pre-treatment:

- participants receiving the assessment interview would have reduced their drinking more than those not receiving the assessment interview; and
- participants receiving the assessment interview would have increased their motivation to change their drinking more than those not receiving the assessment interview.

We further hypothesized that reductions at pre-treatment would be significantly associated with drinking at the 3-month follow-up.

## Methods

### Trial design

This study was a 2X2 factorial randomized controlled trial where all participants received access to an 8-week ICBT program, and either an assessment interview (Factor 1), guidance (Factor 2), a combination of these (Factor 1 and 2), or neither of these. The trial was conducted at the Online Therapy Unit (OTU; www.onlinetherapyuser.ca), based at the University of Regina, Saskatchewan, Canada. The OTU routinely offers ICBT to residents of Saskatchewan free of charge, financed by the Saskatchewan Ministry of Health. The study was registered at www.clinicaltrials.gov (NCT03984786) and approved by the University of Regina Ethics Review Board (approval number 2019-058). The protocol has been published [21].

### Participants

Participants were recruited through a variety of methods, including Google and Facebook ads across Canada; emails and posters distributed to primary care physicians in Saskatchewan; and emails sent to Canadian organizations. Interested participants were directed to the OTU webpage, where they could complete a consent form and questions regarding contact information, background information (e.g., demographics, medical history, mental health history etc.), alcohol use, depression, and anxiety. Applicants meeting initial inclusion criteria scheduled a telephone screening call with OTU staff. In the call (conducted within 1–2 weeks of survey completion), applicants were asked follow-up questions to the screening questions to confirm eligibility, and were asked to verbally consent to participation. To be included in the trial, participants had to (a) be 18 years or older; (b) be a Canadian resident; (c) have access to the internet; (d) score ≥ 8 on the Alcohol Use Disorder Identification Test (AUDIT) [22] indicating at least hazardous drinking; and (e) have consumed ≥ 14 drinks in the preceding week. Applicants were excluded from the trial if they presented with (a) > 24 on the Patient Health Questionnaire 9-item (PHQ-9) [23]; b) suicidal ideation; (c) unmanaged bipolar disorder or schizophrenia; (d) > 24 on the Drug Use Disorder Identification Test (DUDIT) [24] or severe substance use problems as assessed in the telephone enrollment call; (e) low motivation to engage with online treatment as assessed in the telephone enrollment call; (f) frequent visits with a mental health professional (i.e., more than twice a month); or (g) hospitalization for mental health reasons in the past year. Ineligible applicants were referred to appropriate services or were offered the course without being included in the trial.

### Randomization

Immediately after eligibility had been confirmed and verbal consent had been obtained in the telephone screening call, screening staff randomized participants to one of the four treatment conditions. The randomization sequence was pre-generated on http://randomization.com using blocks of 16 and uploaded to the survey system used for data collection. The sequence was hidden from the staff randomizing and participants were blinded to the factors investigated. At the end of screening calls, all participants received a username and temporary password, along with instructions for accessing the course. All participants were given access to the ICBT program on the second Monday after randomization. This treatment delay was chosen to allow a minimum period of nine days to evaluate changes in drinking and motivation to change resulting from the assessment interview.

### Intervention: the alcohol change course

The Alcohol Change Course (ACC) is an ICBT program originally developed in Switzerland [25–27] and translated to English [28] to target alcohol misuse and depression in young adults. For the purpose of this study, the program was adapted for use by adults, with relevant information about alcohol use in Canada (prevalence, guidelines etc.), abstinence, and the impact of alcohol on physical health added to Lesson 1. Further, the program content was restructured to be consistent with the OTU’s other ICBT programs; each lesson consisted of information provided in a slide show format, combined with case stories, worksheets to practice skills, and quizzes and exercises related to each lesson. Participants could download worksheets for use at a later date. The 12 lessons were delivered consecutively over the span of 8 weeks. The adaptation process was completed by a patient-oriented working group consisting of four patients, two clinicians, two managers, two trainees, and two group facilitators. The program was pilot-tested with nine participants before formal start of the factorial trial.

### Experimental factors

#### Factor 1: assessment interview

The assessment interview was administered in the screening call immediately following the randomization. The purpose of the assessment interview was to increase participants’ insight into their own alcohol habits and their consequences through a supportive conversation guided by the AUD module of the Structured Clinical Interview Diagnostic Statistical Manual 5 – Research Version (SCID-5 RV) [29]. In total, three screeners were involved in the screening. The screeners ranged in background training and included Bachelor’s degrees in social work (n = 2) and a Master’s degree in counselling psychology (n = 1). Screeners received a SCID-5 training session as well as training and supervision provided by authors CS and MN. Since the goal of the interview was not to establish a diagnosis, participants were not informed about how many criteria they screened positive for. Screening calls with the assessment interview were 36.0 min (SD = 11.2), while screening calls without the assessment interview were 18.4 min (SD = 6.4), (t = − 14.771, p =  < 0.001, Cohen´s d = 1.87, CI: 1.572–2.169).

#### Factor 2: guidance

Two clinicians provided guidance. Clinicians held graduate degrees in counselling psychology (MEd) and social work (MSW) and had been practicing for 1 and 13 years, respectively, at the time of study onset. They were blinded to whether participants had been randomized to the assessment interview or not and were instructed to spend approximately 15 min per week communicating with each participant, primarily through messages on the treatment platform. In these messages, clinicians were to answer participants’ questions, reinforce module completion, and boost motivation. In rare cases, clinicians contacted participants by telephone (i.e., if a participant requested a call, if there was increased suicide risk, or to address misunderstanding).

Across all four conditions, participants received automated, weekly emails with information about new lesson content. Those randomized to a group without guidance did not receive any other regular contact, but a member of the research team reviewed weekly survey responses to check for significant clinical issues requiring attention (i.e., major increase in drinking, sudden increase in depression symptoms or suicidal ideation). If any participant was deemed at risk, they were contacted and offered referral to appropriate health care. However, they were only discontinued from the trial if they requested it. All participants could contact the OTU if they experienced technical difficulties related to the treatment platform or wanted to discontinue the intervention.

### Monitoring of participants

Each week throughout the 8-week course, all participants who logged in to the platform were asked to complete two questions about past week alcohol use; (1) How many drinks have you had in the past week; and (2) Over how many days did you consume these drinks? They also completed the Patient Health Questionnaire-4 (PHQ-4), a brief questionnaire assessing depression and anxiety [30], and item 9 from PHQ-9 assessing suicidal ideation [23]. These weekly questionnaires were not intended as outcome measures, but allowed systematic monitoring of client symptoms as a safety measure. Participants also responded to reflection questions asking them to list challenges they had with the exercises and to provide examples of what they had learned.

### Measurements

Participants were asked to complete online questionnaires with outcome measures at screening, pre-treatment, mid-treatment (4 weeks into the treatment), post-treatment (8 weeks), and 3-month post-treatment (6 month and 1 year follow-up data collection is ongoing). Participants who did not complete questionnaires were contacted via telephone and/or email as a reminder to complete measures, with a maximum of three reminders per follow-up period. The primary outcomes were number of drinks and number of heavy drinking days (HDD; defined as ≥ 4 drinks per day for women and ≥ 5 drinks per day for men) in the preceding week. Secondary measures were the AUDIT [22], the Penn Alcohol Craving Scale (PACS) [31], and the Brief Situational Confidence Questionnaire (BSCQ) [32]. Additional measures included daily functioning (Sheehan Disability Scale; SDS) [33], depression (the Patient Health Questionnaire-9; PHQ-9) [23], and anxiety (Generalized Anxiety Disorder-7; GAD-7) [34]. Initial motivation, as well as motivational changes between screening and treatment start were assessed using the Readiness to Change Questionnaire –Treatment Version (RCQ-TV) [35]. Finally, treatment credibility was assessed at mid-treatment using the Credibility/Expectancy Questionnaire [36]. Questions pertaining to treatment evaluation and negative effects were assessed at post-treatment and have previously been reported for the overall sample [37]. Treatment engagement was assessed via: number of lessons accessed, days between first and last access to the website, and mean number of website log-ins.

### Sample size and power analysis

The trial aimed to recruit 300 participants to the four conditions (75 participants per group). To estimate this sample size, we used the Factorial PowerPlan provided in the R package MOST [38]. Regarding effect size estimates of factor 1 (assessment interview) we had no available studies to draw on, and therefore pragmatically decided to estimate the effect size as 0.35, as this was the minimum effect size that would indicate this factor to be worth implementing, considering the time spent by staff conducting the interview. Regarding factor 2 (guidance), we estimated the effect size as 0.35, based on previously published studies [7, 8]. Power was set at 80%, alpha at 0.05, and we assumed a correlation of 0.5 between pre-and post-test measurements, and an attrition of 30%

### Statistical analyses

At least one primary or secondary outcome measure was missing for 27% of the sample at post-treatment and 37% at 3-month follow-up. Missing responses were strongly associated with fewer lessons completed (p < 0.001) and with the self-guided course (p = 0.002). To attempt to control for possible bias from differential response rates we used a multiple imputation procedure to replace missing outcome measures. Fifty imputed datasets were created with the MICE package in R [39]. The imputation models controlled for lesson completion, treatment factors, observed values of that measure at other observation times, and interactions between lesson completion, treatment, and observed values.

To evaluate changes in primary and secondary measures, we modeled responses using generalized estimating equations (GEE) [40] using the geepack package, version 1.3.2. in R. The GEE models used Gamma distributions to accommodate skewed response distributions and a log-link function to model changes as proportional to pre-treatment severity. We specified an exchangeable working correlation matrix within individuals to address within-subject correlations, estimated standard errors with robust “sandwich” estimates, and tested whether interactions between Time and Factor were significant using the multivariate Wald test pooling results from the multiply imputed datasets. To evaluate whether the assessment interview had an effect on RCQ-TV change between screening and pre-treatment we planned to use a multinomial logit model. However, as virtually all participants were in the same “readiness to change” stage at screening (i.e., contemplation stage) and at pretreatment (i.e., action stage) respectively, we did not conduct this analysis. To evaluate changes in drinking between screening and pre-treatment, two-way ANOVA’s were used, and to evaluate program engagement, credibility, satisfaction and negative effects by group, one-way ANOVA’s were used. SPSS 25 was used for descriptive statistics and to analyze changes between screening and pre-treatment, while R version 4.1.0. was used for all outcome analyses.

## Results

### Participation flow

**Fig. 1:**
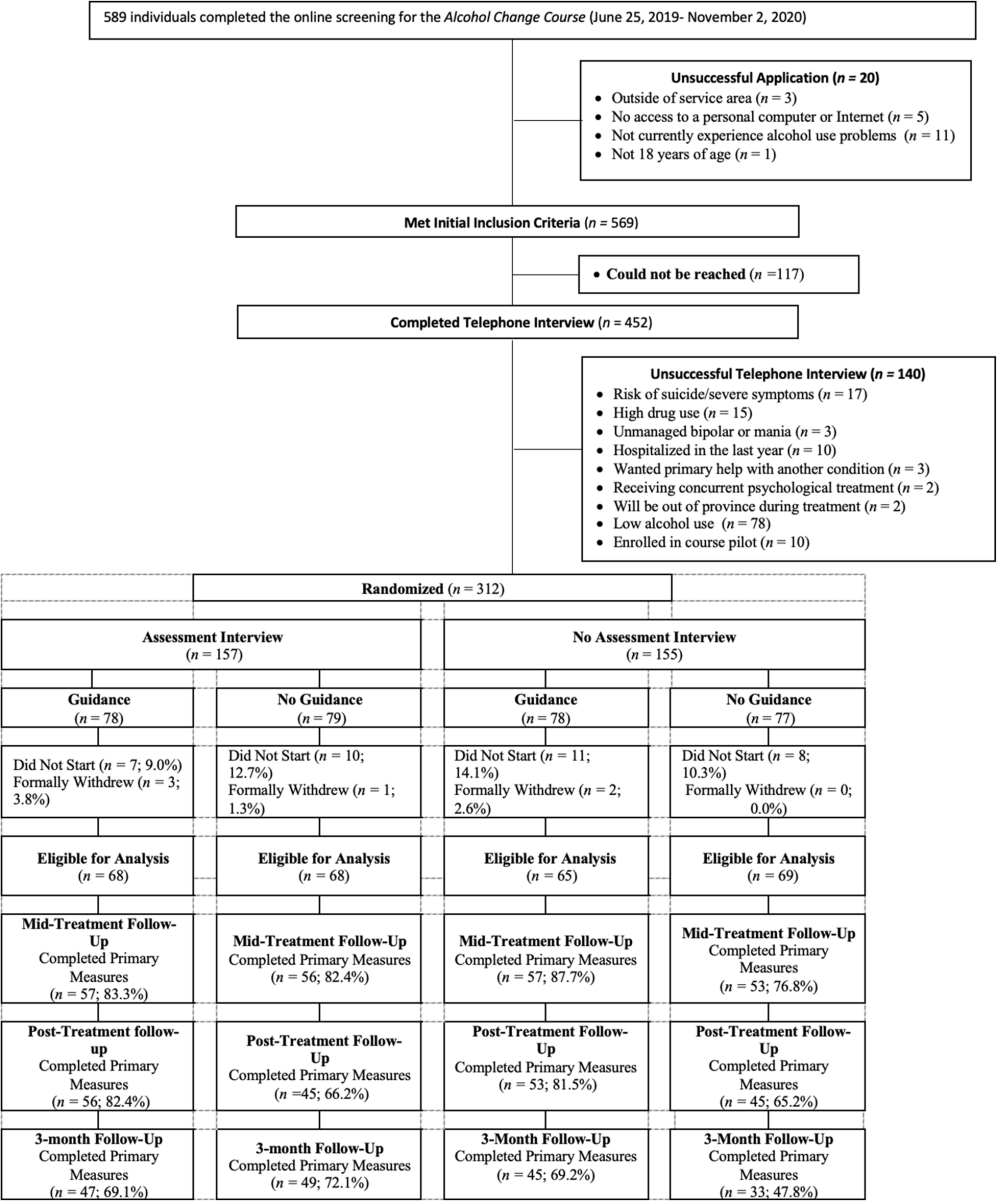
Flow chart

Between August 1, 2019 and November 2, 2020, 312 individuals were randomized. Of these, 36 (11.5%) did not start treatment and 6 (2%) formally withdrew, leaving a sample of 270 participants. See Fig. 1 for overview of trial flow.

### Baseline characteristics

Of the 270 participants, 180 (66.8%) were female and average age was 46.5 (SD = 11.3; range: 22–72), see Table 1. The sample was predominately married (n = 170, 63.0%), employed (n = 187, 69.3%), White (n = 252, 93.3%), and had formal education following high school (n = 231, 85.6%). Participants reported having had 41.7 (SD = 24.8) drinks and 4.5 (SD = 2.1) HDD in the preceding week and the mean AUDIT score was 24.0 (SD = 5.8). No significant differences were identified between groups at baseline.

**Table 1:** Baseline demographic and clinical characteristics

	Randomized participants who began course (N = 270)		Assessment interview (*n* = 136)				No assessment interview (*n* = 134)			
			Guidance (*n* = 68)		No guidance (*n* = 68)		Guidance (*n* = 65)	No guidance (*n* = 69)		
	*n*	%	*n*	%	*n*	%	*n*	%	*n*	%
Age (mean; *SD*)	46.5 (11.3)		46.5 (11.4)		47.7 (11.5)		46.2 (12.0)	45.5 (10.2)		
Gender										
Male	89	33.0	24	35.3	23	33.8	21	32.3	21	30.4
Female	180	66.7	44	64.7	44	64.7	44	67.7	48	69.6
Non-binary	1	0.4	0	0.0	1	1.5	0	0.0	0	0.0
Marital status										
Single/never married	42	15.5	12	17.6	10	14.7	9	13.8	11	15.9
Married/common law	170	63.0	43	63.3	43	63.2	42	64.6	42	60.9
Separated/divorced/widowed	58	21.5	13	19.1	15	22.1	14	21.5	16	23.2
Education										
Less than high school	7	2.6	3	4.4	1	1.5	1	1.5	2	2.9
High school diploma	32	11.9	8	11.8	9	13.2	9	13.8	6	8.7
Post high school certificate/diploma	108	40.0	28	41.2	27	39.7	23	35.4	30	43.5
University education	123	45.5	29	42.6	31	45.6	32	49.2	31	44.9
Employment status										
Employed part-time/full-time	187	69.3	52	76.4	45	66.2	43	66.2	47	68.1
Unemployed	42	15.5	8	11.8	9	13.2	10	15.4	15	21.7
Student, retired, or disability	41	15.2	8	11.8	14	20.6	12	18.4	7	10.2
Ethnicity										
White	252	93.3	62	91.2	63	92.6	62	95.4	65	94.2
Indigenous	7	2.6	2	2.9	3	4.4	0	0.0	2	2.9
Other	11	4.1	4	5.9	2	2.9	3	4.6	2	2.9
Location										
Large city (over 200,000)	124	45.9	31	45.6	35	51.1	30	46.2	28	40.6
Small to medium city	75	27.8	17	25.0	18	26.5	16	24.6	24	34.8
Small rural location	71	26.3	20	29.4	15	22.0	19	29.2	17	24.6
Taking psychotropic medications	118	43.7	28	41.2	29	42.6	30	46.2	31	44.9
Years with alcohol problems										
0–5 years	99	36.6	25	36.8	24	35.3	23	35.4	27	39.1
6–10 years	42	15.6	14	20.6	8	11.8	8	12.3	12	17.4
More than 10 years	129	47.8	29	42.6	36	52.9	34	52.3	30	43.5
Previous treatment for alcohol problems										
Yes	84	31.1	21	30.9	29	42.6	15	23.1	19	27.5
Type of treatment received previously										
Alcoholics anonymous	46	17.0	13	19.1	16	23.5	6	9.2	11	15.9
Individual psychotherapy or counselling	57	21.1	14	20.6	20	29.4	12	18.5	11	15.9
Group psychotherapy	12	4.4	5	7.4	4	5.9	2	3.1	1	1.4
Medical treatment	23	8.5	6	8.8	7	10.3	4	6.2	6	8.7
Self-help (Books, Online forums)	4	1.5	1	1.5	1	1.5	0	0.0	2	2.9

	Mean	SD	Mean	SD	Mean	SD	Mean	SD	Mean	SD
Drinks preceding week	41.7	24.8	40.9	24.4	41.2	26.0	39.2	18.7	45.5	29.0
Heavy drinking days preceding week	4.5	2.1	4.4	2.1	4.3	2.2	4.6	2.2	4.7	2.1
Alcohol Use Disorder Identification Test (AUDIT)	24.0	5.8	24.0	5.6	24.2	5.6	23.8	5.7	24.2	6.3
Patient Health Questionnaire (PHQ-9)	10.8	6.0	10.3	5.5	10.6	6.1	10.5	6.2	11.6	6.2
Generalized Anxiety Disorder-7 (GAD-7)	8.6	5.9	8.4	6.0	8.4	5.8	8.5	5.9	8.9	6.1
Penn Alcohol Craving Scale (PACS)	17.8	5.7	17.4	5.6	18.0	6.0	17.5	6.0	18.3	5.3
Drug Use Disorder Identification Test (DUDIT)	2.4	4.4	2.6	4.8	3.0	5.2	1.4	3.5	2.4	3.9
Brief Situational Confidence Questionnaire (BSCQ)	321.5	147.6	305.8	146.8	339.9	161.8	312.9	143.2	327.2	138.3
Sheehan Disability Scale (SDS)	15.6	7.9	15.2	7.9	15.5	8.1	14.5	7.9	16.9	7.5

### Changes between screening and pre-treatment

#### Readiness to change

Nearly all patients had moved to the action stage by pre-treatment, see Table 2.

**Table 2:** Screening and pre-treatment by group (assessment interview vs no Assessment interview): Readiness to change

	Screening				Pre-treatment			
Stage	Assessment interview		No assessment interview		Assessment interview		No assessment interview	
	n	%	n	%	n	%	n	%
Precontemplation	1	0.7	0	0	1	0.7	1	0.7
Contemplation	116	85.2	115	85.8	2	1.5	2	1.5
Action	19	14.0	19	14.2	133	97.8	131	97.8
Total	136	100	134	100	136	100	134	100

#### Alcohol consumption

Overall reductions were observed between screening and at pre-treatment, but there were no significant differences between the groups in either drinks or HDD (see Table 3). Fifteen participants in each group (11.0% vs 11.2%) reported being abstinent at pre-treatment.

**Table 3:** Screening and pre-treatment by group (assessment interview vs no assessment interview): Alcohol consumption

Primary Outcomes	Screening		Pre-treatment		Time effect*	Group effect*	Interaction*
	*M*	SD	*M*	SD			
Drinks							
Assessment interview	41.1	25.1	29.9	24.9	*F*_(1,268)_ = 61.30, *p* < .0001	*F*_(1,268)_ = 0.11, *p* = .74	*F*_(1, 268)_ = 0.08, *p* = .78
No assessment interview	42.3	24.6	30.6	22.1			
Heavy Drinking Days							
Assessment interview	4.4	2.1	3.3	2.4	*F*_(1,268)_ = 86.17, *p* < .0001	*F*_(1,268)_ = 2.40, *p* = .12	*F*_(1, 268)_ = 1.38, *p* = .24
No assessment interview	4.6	2.11	3.6	2.4			

### Primary outcomes

All groups showed large within-group reductions in drinks and HDD from screening to post-treatment and from screening to 3-month follow-up (Cohen’s *d*_*w*_ ≥ 0.82, proportional reduction ≥ 48%, *p* < 0.001). The Time*factor interactions of reductions in drinks and HDD for pre-treatment to post-treatment and to the 3-month follow-up were not significant for either treatment factor (*p* > 0.07), and neither were the overall tests of time*factor (drinks; assessment interview: *p* = 0.29, guidance *p* = 0.50, HDD; assessment interview: *p* = 0.56, guidance: *p* = 0.65). See Table 4 for findings per group and Additional file 1: Table S1 for findings by factor. The combination of factors (assessment interview + guidance) did not appear to render greater alcohol reductions. However, alcohol reductions from screening to pre-treatment significantly correlated with improvements from screening to 3-month follow-up on both drinks (*r* = 0.52, *p* < 0.001) and HDD (*r* = 0.46, *p* < 0.001).

**Table 4:** Estimated marginal means, within-group effect sizes and proportional reductions at all time points by group^*^

	Factor 1: Assessment interview	Factor 2: Guidance	Screening	Pre-treatment	Mid-treatment	Post-treatment	3 month follow-up	Within-group effect size (Cohen’s d) with confidence intervals				% change			
								Pre-treatment	Mid-treatment	Post-treatment	3 month follow-up	Pre-treatment	Mid-treatment	Post-treatment	3 month follow-up
Reductions in drinks	Assessment interview	Guidance	40.9 (24.4)	30.1 (26.8)	20.4 (21.3)	17.9 (19.1)	21.0 (22.8)	0.42 [0.08, 0.76]	0.89 [0.54, 1.24]	1.05 [0.69, 1.41]	0.84 [0.49, 1.19]	26.4 [10.9, 41.9]	50.1 [37.4, 62.7]	56.3 [44.2, 68.5]	48.7 [34.3, 63.1]
		No guidance	41.2 (26.0)	29.8 (23.0)	23.5 (27.0)	18.7 (22.2)	20.1 (26.4)	0.46 [0.12, 0.80]	0.66 [0.32, 1.01]	0.92 [0.57, 1.28]	0.80 [0.45, 1.15]	27.7 [14.5, 40.9]	43.0 [26.9, 59.1]	54.6 [40.6, 68.7]	51.1 [35.0, 67.2]
	No assessment interview	Guidance	39.2 (18.7)	30.06 (19.8)	17.8 (15.1)	14.6 (15.2)	16.6 (18.3)	0.47 [0.12, 0.82]	1.25 [0.88, 1.63]	1.43 [1.05, 1.82]	1.21 [0.84, 1.59]	23.2 [11.0, 35.4]	54.7 [44.9, 64.5]	62.7 [52.6, 72.8]	57.7 [45.6, 69.7]
		No guidance	45.5 (29.0)	31.16 (24.2)	21.8 (22.1)	15.4 (17.3)	21.6 (22.0)	0.53 [0.20, 0.87]	0.92 [0.57, 1.27]	1.26 [0.89, 1.62]	0.93 [0.57, 1.28]	31.5 [19.1, 44.0]	52.1 [39.7, 64.5]	66.2 [56.1, 76.3]	52.6 [39.1, 66.1]
Reductions in HDD	Assessment interview	Guidance	4.4 (2.1)	3.3 (2.4)	2.2 (2.4)	2.0 (2.3)	1.9 (2.5)	0.49 [0.15, 0.84]	0.99 [0.63, 1.34]	1.12 [0.76, 1.48]	1.10 [0.74, 1.46]	24.9 [12.2, 37.6]	50.4 [36.8, 64.0]	55.7 [42.2, 69.2]	56.4 [41.7, 71.2]
		No guidance	4.3 (2.2)	3.2 (2.4)	2.2 (2.4)	1.8 (2.3)	2.1 (2.6)	0.46 [0.12, 0.80]	0.89 [0.54, 1.24]	1.10 [0.74, 1.46]	0.92 [0.56, 1.27]	24.7 [11.5, 37.9]	48.3 [33.9, 62.7]	58.3 [44.3, 72.3]	51.4 [35.6, 67.3]
	No assessment interview	Guidance	4.6 (2.2)	3.8 (2.3)	2.2 (2.2)	1.5 (1.8)	1.8 (2.1)	0.37 [0.02, 0.71]	1.09 [0.72, 1.46]	1.57 [1.18, 1.96]	1.31 [0.93, 1.69]	18.1 [6.0, 30.3]	52.7 [40.4, 65.1]	68.3 [58.3, 78.3]	61.2 [48.8, 73.6]
		No guidance	4.7 (2.1)	3.5 (2.6)	2.3 (2.4)	1.9 (2.3)	2.4 (2.5)	0.49 [0.15, 0.83]	1.06 [0.70, 1.42]	1.24 [0.88, 1.61]	0.97 [0.62, 1.33]	24.6 [11.7, 37.5]	51.6 [38.1, 65.2]	58.9 [45.7, 72.2]	48.6 [33.0, 64.3]
AUDIT	Assessment interview	Guidance	23.00 (5.55)	–	–	16.70 (8.04)	15.08 (9.40)	–	–	0.91 [0.55, 1.26]	1.02 [0.66, 1.38]	–	–	27.4 [18.5, 36.2]	34.5 [23.6, 45.3]
		No guidance	23.15 (5.61)	–	–	16.21 (7.39)	13.28 (7.87)	–	–	1.05 [0.69, 1.41]	1.44 [1.06, 1.81]	–	–	30.0 [20.8, 39.1]	42.6 [33.5, 51.8]
	No assessment interview	Guidance	22.77 (5.72)	–	–	15.60 (7.34)	14.02 (7.50)	–	–	1.08 [0.72, 1.45]	1.30 [0.93, 1.68]	–	–	31.5 [22.8, 40.2]	38.4 [29.6, 47.3]
		No guidance	23.16 (6.29)	–	–	15.59 (7.80)	14.39 (8.10)	–	–	1.06 [0.71, 1.42]	1.20 [0.84, 1.57]	–	–	32.7 [23.4, 42.0]	37.9 [26.4, 49.3]
PACS	Assessment interview	Guidance	17.43 (5.59)	–	–	12.45 (6.25)	11.87 (7.44)	–	–	0.83 [0.48, 1.19]	0.84 [0.49, 1.19]	–	–	28.6 [19.3, 37.8]	31.9 [20.6, 43.1]
		No guidance	18.04 (6.02)	–	–	12.91 (6.63)	10.74 (6.75)	–	–	0.81 [0.46, 1.15]	1.14 [0.77, 1.50]	–	–	28.4 [18.5, 38.4]	40.5 [30.7, 50.3]
	No assessment interview	Guidance	17.46 (5.99)	–	–	11.59 (6.61)	10.29 (6.11)	–	–	0.93 [0.56, 1.29]	1.18 [0.81, 1.55]	–	–	33.6 [23.9, 43.4]	41.1 [31.0, 51.1]
		No guidance	18.33 (5.33)	–	–	12.76 (6.75)	12.64 (6.94)	–	–	0.91 [0.56, 1.26]	0.91 [0.56, 1.27]	–	–	30.4 [20.5, 40.2]	31.1 [19.6, 42.5]
BSCQ	Assessment interview	Guidance	305.78 (146.75)	–	–	474.28 (177.31)	459.26 (195.69)	–	–	1.03 [0.67, 1.39]	0.88 [0.53, 1.23]	–	–	34.1 [25.0, 43.2]	31.1 [20.2, 41.9]
		No guidance	339.85 (161.78)	–	–	480.16 (181.81)	489.38 (186.59)	–	–	0.81 [0.46, 1.16]	0.85 [0.50, 1.20]	–	–	30.5 [19.3, 41.7]	32.5 [21.8, 43.2]
	No assessment interview	Guidance	312.89 (143.22)	–	–	529.65 (164.54)	512.06 (175.51)	–	–	1.40 [1.01, 1.78]	1.24 [0.86, 1.61]	–	–	44.5 [35.8, 53.2]	40.9 [30.2, 51.6]
		No guidance	327.19 (138.34)	–	–	505.93 (175.00)	538.94 (179.40)	–	–	1.13 [0.77, 1.49]	1.31 [0.95, 1.68]	–	–	37.8 [27.9, 47.7]	44.8 [33.8, 55.8]
SDS	Assessment interview	Guidance	15.16 (7.94)	–	–	8.46 (7.91)	5.83 (7.23)	–	–	0.84 [0.49, 1.19]	1.22 [0.86, 1.59]	–	–	44.2 [30.9, 57.6]	61.5 [47.4, 75.6]
		No guidance	15.49 (8.07)	–	–	7.04 (7.77)	6.06 (7.69)	–	–	1.06 [0.70, 1.42]	1.19 [0.82, 1.55]	–	–	54.6 [40.1, 69.0]	60.9 [47.5, 74.3]
	No assessment interview	Guidance	14.54 (7.91)	–	–	6.86 (8.31)	4.52 (6.49)	–	–	0.94 [0.58, 1.30]	1.38 [0.99, 1.76]	–	–	52.8 [38.4, 67.2]	68.9 [55.3, 82.5]
		No guidance	16.94 (7.53)	–	–	6.41 (7.93)	7.80 (8.20)	–	–	1.36 [0.99, 1.73]	1.16 [0.79, 1.52]	–	–	62.2 [49.4, 75.0]	54.0 [37.8, 70.1]
PHQ-9	Assessment interview	Guidance	10.31 (5.46)	–	6.09 (4.48)	5.65 (4.87)	5.88 (5.12)	–	0.84 [0.49, 1.19]	0.89 [0.54, 1.25]	0.83 [0.48, 1.18]	–	40.9 [29.7, 52.1]	45.2 [33.0, 57.3]	43.0 [29.4, 56.6]
		No guidance	10.59 (6.12)	–	7.22 (5.86)	5.91 (4.86)	5.05 (4.01)	–	0.56 [0.22, 0.90]	0.84 [0.49, 1.19]	1.06 [0.70, 1.42]	–	31.8 [17.9, 45.7]	44.1 [31.0, 57.3]	52.3 [41.6, 62.9]
	No assessment interview	Guidance	10.54 (6.23)	–	5.63 (4.56)	5.16 (4.37)	5.96 (5.41)	–	0.90 [0.53, 1.26]	0.99 [0.63, 1.36]	0.78 [0.42, 1.14]	–	46.6 [35.4, 57.8]	51.1 [40.0, 62.1]	43.4 [29.5, 57.4]
		No guidance	11.55 (6.23)	–	6.45 (5.11)	6.71 (5.28)	6.50 (5.74)	–	0.89 [0.54, 1.24]	0.83 [0.49, 1.18]	0.84 [0.49, 1.19]	–	44.2 [32.8, 55.6]	41.9 [29.7, 54.1]	43.7 [29.2, 58.2]
GAD-7	Assessment interview	Guidance	8.37 (6.01)	–	–	4.78 (4.89)	4.85 (4.78)	–	–	0.65 [0.31, 1.00]	0.64 [0.30, 0.99]	–	–	42.9 [27.9, 57.8]	42.0 [26.5, 57.6]
		No guidance	8.44 (5.78)	–	–	4.70 (3.90)	4.72 (4.03)	–	–	0.76 [0.41, 1.10]	0.74 [0.39, 1.09]	–	–	44.4 [30.3, 58.4]	44.1 [30.8, 57.4]
	No assessment interview	Guidance	8.45 (5.88)	–	–	4.61 (4.29)	4.79 (5.53)	–	–	0.74 [0.39, 1.10]	0.64 [0.28, 0.99]	–	–	45.5 [32.2, 58.7]	43.2 [26.1, 60.4]
		No guidance	8.93 (6.12)	–	–	5.89 (4.67)	5.28 (4.15)	–	–	0.55 [0.21, 0.89]	0.69 [0.35, 1.04]	–	–	34.0 [19.1, 48.8]	40.8 [25.3, 56.4]

*HDD* heavy drinking days, *AUDIT* Alcohol Use Disorder Identification Test, *PACS* Penn Alcohol Craving Scale, *BSCQ* Brief Situational Confidence Questionnaire, *SDS* Sheehan Disability Scale, *PHQ-9* Patient Health Questionnaire, *GAD-7* Generalized Anxiety Disorder

^*^Marginal means were estimated using GEE models. Cohen's d effect sizes and % changes in outcome measures were calculated from the marginal means

### Secondary outcomes

There were large within-group improvements on AUDIT, PACS, BSCQ, SDS, and PHQ-9 (Cohen’s *d*_*w*_ ≥ 0.81, proportional reduction ≥ 27%, p < 0.001), and moderate improvements on the GAD-7 (Cohen’s *d*_w_ ≥ 0.55, proportional reduction ≥ 34%, *p* < 0.001), see Table 4. One significant time*factor interaction was found on the BSCQ, where the group receiving the assessment interview had smaller improvements from screening to 3-month follow-up (31.8%) than the group with no assessment interview (42.9%, *p* = 0.04). The overall test of time*assessment interview interactions for the BSCQ, however, was not significant (*p* = 0.09), see Additional file 2: Table S2. There were no significant time*factor interactions on any other secondary measure (*p* > 0.05) and neither were there any significant overall tests of time*factor.

Participants completed an average of 8.3 lessons (*SD* = 3.8), with those receiving guidance completing significantly more lessons (*p* < *0.001)*. Those who received guidance were significantly more likely to use the program for a longer duration (*p* = *0.01)* and had significantly higher number of log-ins (*p* < *0.001)*, see Table 5.

**Table 5:** Program engagement, credibility, satisfaction and negative effects by group

			Assessment interview (*n* = 136)				No assessment interview (*n* = 134)					
	All Groups (N = 270) Mean (*SD*)		Guidance (*n* = 68) Mean (*SD*)		No guidance (*n* = 68) Mean (*SD*)		Guidance (*n* = 65) Mean (*SD*)		No guidance (*n* = 69) Mean (*SD*)		Statistical Significance	
											Interview	Guidance
Number of completed modules	8.3 (3.8)		8.8 (3.6)		8.0 (3.8)		9.4 (3.4)		6.8 (4.0)		*F*_(1, 269)_ = 0.38, *p* = .54	*F*_(1, 269)_ = 14.41, *p* < .001
											Interaction: *F*_(1, 269)_ = 3.73, *p* = 0.05	
Days between first and last log-in	46.8 (29.2)		49.9 (26.0)		42.5 (30.7)		52.6 (24.7)		42.6 (33.7)		*F*_(1, 269)_ = 0.15, *p* = .70	*F*_(1, 269)_ = 6.06, *p* = .01
											Interaction: *F*_(1,269)_ = .14, *p* = .71	
Number of log-ins	14.5 (10.5)		16.9 (10.2)		13.6 (10.0)		17.4 (13.7)		10.4 (5.2)		*F*_(1, 269)_ = 1.18, *p* = .28	*F*_(1, 269)_ = 17.43, *p* < .001
											Interaction: *F*_(1,269)_ = 2.26, *p* = .13	
Written messages sent clinician to client	4.2 (4.1)		8.1 (1.7)		0.2 (0.6)		8.3 (1.2)		0.3 (0.6)		*F*_(1, 269)_ = 1.28, *p* = .26	*F*_(1, 269)_ = 3474.30, *p* < .001
											Interaction: *F*_(1,269)_ = 0.52, *p* = .47	
Written messages sent from client to clinician	1.5 (2.1)		2.6 (2.5)		0.5 (1.2)		2.5 (2.4)		0.4 (0.8)		*F*_(1, 269)_ = 0.06, *p* = .80	*F*_(1, 269)_ = 85.90, *p* < .001
											Interaction: *F*_(1,269)_ = 0.01, *p* = .92	
Number of phone calls with clinician	0.8 (1.0)		0.9 (1.3)		0.5 (0.8)		0.9 (1.1)		0.7 (0.8)		*F*_(1, 269)_ = 0.51, *p* = .48	*F*_(1, 269)_ = 5.96, *p* = .02
											Interaction: *F*_(1,269)_ = 0.22, *p* = .88	
Midtreatment credibility (6–27)	21.39 (4.48)		20.93 (4.50)		21.29 (4.83)		21.85 (4.54)		21.49 (4.06)		*F*_(1, 219)_ = 0.87, *p* = .35	*F*_(1, 219)_ = 0.00, *p* = .99
											Interaction: *F*_(1,219)_ = 0.35, *p* = .55	
Midtreatment expectancy (1–29)	19.76 (5.88)		19.59 (6.31)		19.25 (5.84)		20.05 (6.07)		20.17 (5.34)		*F*_(1, 219)_ = 0.76, *p* = .39	*F*_(1, 219)_ = 0.02, *p* = .89
											Interaction: *F*_(1,219)_ = 0.08, *p* = .78	
Satisfied/very satisfied overall, *n* (%)	133	69.6	36	67.9	28	62.2	39	75.0	30	73.2	*X*^2^_(1, 191),_ = 1.77; *p* = .18	*X*^2^_(1, 191),_ = 0.35; *p* = .55
											Interaction: *X*^2^_(1, 191),_ = 0.06; *p* = .81	
Satisfied/very satisfied with materials, *n* (%)	154	80.6	42	79.2	36	80.0	43	82.7	33	80.5	*X*^2^_(1, 191),_ = 0.14; *p* = .71	*X*^2^_(1, 191),_ = 0.02; *p* = .90
											Interaction: *X*^2^_(1, 191),_ = 0.07; *p* = .79	
Increased/greatly increased confidence, *n* (%)	156	81.7	42	79.2	35	77.8	47	90.4	32	78.0	*X*^2^_(1, 191),_ = 1.30; *p* = .26	*X*^2^_(1, 191),_ = 1.48; *p* = .22
											Interaction: *X*^2^_(1, 191),_ = 1.29; *p* = .26	
Increased/greatly increased motivation for other treatment, *n* (%)	149	78.0	43	81.1	37	82.2	38	73.1	31	75.6	*X*^2^_(1, 191),_ = 1.54; *p* = .22	*X*^2^_(1, 191),_ = 0.10; *p* = .75
											Interaction: *X*^2^_(1, 191),_ = 0.01; *p* = .93	
Course was worth the time, *n* (%)	180	94.2	49	92.5	42	93.3	50	96.2	39	95.1	*X*^2^_(1, 191),_ = 0.71; *p* = .40	*X*^2^_(1, 191),_ = 0.001; *p* = .98
											Interaction: *X*^2^_(1, 191),_ = 0.09; *p* = .77	
Would recommend the course to a friend, *n* (%)	179	93.7	51	96.2	40	88.9	50	96.2	38	92.7	*X*^2^_(1, 191),_ = 0.25; *p* = .62	*X*^2^_(1, 191),_ = 2.42; *p* = .12
											Interaction: *X*^2^_(1, 191),_ = 0.14; *p* = .71	
Reported negative effects, *n* (%)	5	2.6	1	1.9	2	4.4	1	1.9	1	2.4	*X*^2^_(1, 191),_ = 0.16; *p* = .69	*X*^2^_(1, 191),_ = 0.46; *p* = .50
											Interaction: *X*^2^_(1, 191),_ = 0.14; *p* = .74	
Negative effect impact (1–4)	2.60 (1.14)	–	2.00 (0.00)	–	2.00 (1.41)	–	3.00 (0.00)	–	4.00 (0.00)	-	*F*_(1,4)_ = 0.47, *p* = .76	*F*_(1,4)_ = 0.47, *p* = .76
											Interaction: *F*_(1,4)_ = 0.14, *p* = .77	

### Treatment credibility, satisfaction and negative effects

There were no significant differences among groups in treatment credibility, satisfaction, or negative effects (See Table 5). The majority of participants indicated that they were either ‘Satisfied’ or ‘Very satisfied’ with overall treatment (*n* = 136, 70.1%) and treatment materials (*n* = 155, 80.0%). Few patients reported negative or adverse events resulting from participation in the ACC (2.6%, *n* = 6).

## Discussion

The aim of this randomized factorial trial was to examine ways to optimize delivery of ICBT for alcohol misuse in a routine online therapy clinic by examining effects of two factors: (1) a pre-treatment assessment interview and (2) guidance. Large within-group reductions were observed in all groups, but no main effects were observed for the two factors and neither did we observe an interaction effect. Similarly, moderate to large within-group effect sizes were found for secondary outcomes but no effects related to the two factors. As for initial change (between screening and pre-treatment), significant within-group reductions in both groups were observed, and nearly all participants moved from the contemplation stage to the action stage, regardless of whether they had received the assessment interview or not. Initial reductions in drinks and heavy drinking days correlated significantly with reductions at post-treatment and follow-up.

This is the first time that the effects of a pre-treatment assessment interview has been experimentally investigated in ICBT for alcohol misuse, and it did not improve outcomes in our study. Guidance did not improve outcomes either, which adds to recent studies failing to find differential effects [9–12]. One explanation for the lack of effects may be that the Online Therapy Unit, the clinic where the trial was conducted, by default provides some degree of human contact to all participants regardless of group assignment, something which could potentially increase engagement for everyone [41]. Specifically, the assessment interview was provided as an extension of a screening call that all participants received and may not have been sufficient to produce any additional effects over and above the screening call. Further, in line with routine procedures at the clinic, all participants were called by staff whenever concern about a major increase in alcohol consumption or suicidal ideation was noted in the weekly questionnaires. A relatively large proportion of those randomized to a group without guidance were contacted by staff, possibly contributing to a dilution of the effect of the guidance factor. As has been suggested in research on ICBT for depression and anxiety, it may be that optimal outcomes are achieved when high quality treatment material is combined with clinical interviews and clinical monitoring during treatment [42].

There are limitations to this study. We had missing data for 27% of participants on at least one primary or secondary measure at post-treatment and for 37% of participants at 3-month follow-up. Importantly, we also had differential attrition such that those receiving guidance were significantly more likely to complete both post-treatment and 3-month follow-ups. Although a multiple imputation analysis was conducted to account for potential bias in the data that was collected, it is possible that participants who did not complete the follow-ups differed in other important ways from those who did complete the follow-ups. Although no trends were apparent in the data, it is possible that with a larger sample size, small effects between factors may have been detected. Further, much of the data was collected after the COVID-19 pandemic began in early 2020, something which may have impacted the results. According to Canadian statistics, the pandemic has been associated with increased alcohol use among the one-third of Canadian residents with a history of alcohol use [43] with residents reporting that factors such as stress, boredom and reduced mental health contributed to increased use [44].

Even though the assessment interview did not increase alcohol reductions or initial motivation, the finding that initial reductions (i.e. between screening and pre-treatment) correlated with reductions at follow-ups suggests that initial reductions (or lack thereof) can predict long-term change among clients using these kinds of interventions. Other ways to optimize effects of internet interventions for alcohol misuse, such as post-treatment boosters [45] should be further investigated. Future research may also examine ways to improve engagement and treatment completion.

Most participants moved from the contemplation stage in the screening to the action stage at pre-treatment, but motivation may have waned over the course of the treatment. Interventions that target low motivation when it arises in treatment, such as motivational interviewing (MI) may be beneficial [46] and further research could investigate ways to assess motivation during treatment and offer support when motivation wanes. In a meta-analysis on the treatment of comorbid AUD and depression, combined CBT and MI resulted in small but clinically significant benefits compared to treatment as usual [47]. Lastly, including additional resources related to the use of other substances (e.g., cannabis), may be beneficial to investigate, as individuals with AUD often have comorbid concerns [48].

## Conclusions

ICBT for alcohol misuse was associated with large reductions in alcohol consumption when delivered in a routine online therapy clinic but neither a pre-treatment assessment interview nor guidance from a clinician appeared to increase these reductions.

## Supplementary Information

**Additional file 1: Table S1.** Factor table.

**Additional file 2:** **Table S2.** Overall tests.

## Data Availability

The datasets used and/or analysed during the current study are available from the corresponding author on reasonable request.

## Abbreviations

IPDMA: Individual patient data meta-analysis
PNF: Personalized normative feedback
CBT: Cognitive behaviour therapy
ICBT: Internet-delivered cognitive behaviour therapy
AR: Assessment reactivity
SCID-5: Structured Clinical Interview DSM-5
OTU: Online therapy unit
ACC: Alcohol change course
AUD: Alcohol use disorders
AUDIT: Alcohol Use Disorder Identification Test
DUDIT: Drug Use Disorder Identification Test
PHQ-9: Patient Health Questionnaire 9-item
PHQ-4: Patient Health Questionnaire 4-item
GAD-7: Generalized anxiety disorder-7
HDD: Heavy drinking days
BSCQ: Brief Situational Confidence Questionnaire
SDS: Sheehan disability scale
RCQ-TV: Readiness to change questionnaire–treatment version
GEE: Generalized estimating equations
