# Supplementary material for: Optimizing internet-delivered cognitive behaviour therapy for alcohol misuse—a randomized factorial trial examining effects of a pre-treatment assessment interview and guidance
Source: Addict Sci Clin Pract. 2022 Jul 23;17:37. doi: 10.1186/s13722-022-00319-0 (PMC9308037; doi:10.1186/s13722-022-00319-0)
Supplement: Supplementary file 1 — Additional file 1: Table S1. Factor table. [file 13722_2022_319_MOESM1_ESM.docx]

Additional file 1. Factor table

|  |  | Screening | Pre-treatment | Mid-treatment | Post-treatment | Screening to post-treatment | 3 month follow-up | Screening to 3-month follow-up | Effect size (95% CI) | | | |
| --- | --- | --- | --- | --- | --- | --- | --- | --- | --- | --- | --- | --- |
|  |  | M (sd) | M (sd) | M (sd) | M (sd) | p-values | M (sd) | p-values | Pre-treatment | Mid-treatment | Post-treatment | 3-month follow-up |
| drinks | No guidance | 43.29 (27.52) | 30.45 (23.53) | 22.61 (24.61) | 16.96 (19.94) | 0.8172 | 20.84 (24.26) | 0.8075 | 0.50 [0.26, 0.74] | 0.79 [0.54, 1.04] | 1.09 [0.84, 1.35] | 0.86 [0.62, 1.11] |
| drinks | Guidance | 40.03 (21.72) | 30.09 (23.59) | 19.04 (18.54) | 16.15 (17.32) |  | 18.65 (20.82) |  | 0.44 [0.19, 0.68] | 1.04 [0.78, 1.29] | 1.21 [0.95, 1.47] | 1.00 [0.75, 1.26] |
| drinks | No assessment interview | 42.21 (24.64) | 30.61 (22.11) | 19.66 (19.09) | 14.99 (16.28) | 0.0748 | 18.90 (20.41) | 0.3946 | 0.49 [0.25, 0.74] | 1.02 [0.77, 1.27] | 1.30 [1.04, 1.56] | 1.03 [0.77, 1.28] |
| drinks | Assessment interview | 41.05 (25.11) | 29.94 (24.90) | 21.90 (24.31) | 18.27 (20.68) |  | 20.56 (24.65) |  | 0.44 [0.20, 0.68] | 0.77 [0.53, 1.02] | 0.99 [0.74, 1.24] | 0.82 [0.57, 1.07] |
| HDD | No guidance | 4.46 (2.14) | 3.36 (2.47) | 2.23 (2.41) | 1.85 (2.30) | 0.8519 | 2.23 (2.54) | 0.2159 | 0.47 [0.23, 0.71] | 0.98 [0.72, 1.23] | 1.17 [0.92, 1.43] | 0.95 [0.70, 1.20] |
| HDD | Guidance | 4.50 (2.11) | 3.53 (2.35) | 2.18 (2.32) | 1.69 (2.08) |  | 1.85 (2.27) |  | 0.43 [0.19, 0.68] | 1.04 [0.79, 1.30] | 1.34 [1.07, 1.61] | 1.21 [0.95, 1.47] |
| HDD | No assessment interview | 4.62 (2.12) | 3.63 (2.44) | 2.21 (2.32) | 1.67 (2.07) | 0.2638 | 2.06 (2.34) | 0.5268 | 0.43 [0.19, 0.67] | 1.08 [0.83, 1.34] | 1.41 [1.14, 1.67] | 1.14 [0.89, 1.40] |
| HDD | Assessment interview | 4.35 (2.13) | 3.27 (2.38) | 2.20 (2.42) | 1.87 (2.31) |  | 2.00 (2.49) |  | 0.48 [0.24, 0.72] | 0.94 [0.69, 1.19] | 1.12 [0.86, 1.37] | 1.01 [0.76, 1.26] |
| AUDIT | No guidance | 23.15 (5.94) | - | - | 15.90 (7.59) | 0.6706 | 13.82 (8.00) | 0.4355 | - | - | 1.06 [0.81, 1.31] | 1.32 [1.06, 1.58] |
| AUDIT | Guidance | 22.88 (5.61) | - | - | 16.14 (7.70) |  | 14.54 (8.52) |  | - | - | 1.00 [0.74, 1.25] | 1.15 [0.89, 1.41] |
| AUDIT | No assessment interview | 22.96 (6.00) | - | - | 15.60 (7.56) | 0.4467 | 14.20 (7.81) | 0.9009 | - | - | 1.08 [0.82, 1.33] | 1.25 [0.99, 1.52] |
| AUDIT | Assessment interview | 23.07 (5.56) | - | - | 16.45 (7.70) |  | 14.15 (8.69) |  | - | - | 0.98 [0.73, 1.23] | 1.22 [0.96, 1.48] |
| PACS | No guidance | 18.19 (5.67) | - | - | 12.84 (6.68) | 0.6809 | 11.65 (6.91) | 0.9021 | - | - | 0.86 [0.61, 1.11] | 1.03 [0.78, 1.28] |
| PACS | Guidance | 17.44 (5.76) | - | - | 12.01 (6.42) |  | 11.05 (6.85) |  | - | - | 0.89 [0.64, 1.14] | 1.01 [0.75, 1.26] |
| PACS | No assessment interview | 17.89 (5.66) | - | - | 12.16 (6.69) | 0.4187 | 11.41 (6.65) | 0.9685 | - | - | 0.92 [0.67, 1.17] | 1.05 [0.79, 1.30] |
| PACS | Assessment interview | 17.73 (5.80) | - | - | 12.68 (6.43) |  | 11.29 (7.11) |  | - | - | 0.82 [0.58, 1.07] | 0.99 [0.74, 1.24] |
| BSCQ | No guidance | 333.56 (150.01) | - | - | 493.32 (178.55) | 0.2410 | 515.24 (184.35) | 0.6042 | - | - | 0.97 [0.72, 1.22] | 1.08 [0.82, 1.33] |
| BSCQ | Guidance | 309.35 (144.53) | - | - | 503.26 (172.90) |  | 486.77 (187.62) |  | - | - | 1.21 [0.95, 1.47] | 1.06 [0.80, 1.31] |
| BSCQ | No assessment interview | 320.09 (140.38) | - | - | 518.04 (170.00) | 0.0527 | 525.83 (177.79) | 0.0409^*^ | - | - | 1.27 [1.00, 1.53] | 1.28 [1.02, 1.54] |
| BSCQ | Assessment interview | 323.12 (154.83) | - | - | 477.23 (179.14) |  | 474.67 (191.33) |  | - | - | 0.92 [0.67, 1.17] | 0.87 [0.62, 1.12] |
| SDS | No guidance | 16.20 (7.81) | - | - | 6.71 (7.85) | 0.1240 | 6.87 (8.00) | 0.2668 | - | - | 1.21 [0.95, 1.47] | 1.18 [0.92, 1.43] |
| SDS | Guidance | 14.85 (7.90) | - | - | 7.62 (8.12) |  | 5.14 (6.91) |  | - | - | 0.90 [0.65, 1.15] | 1.31 [1.04, 1.57] |
| SDS | No assessment interview | 15.69 (7.78) | - | - | 6.63 (8.10) | 0.2162 | 5.94 (7.61) | 0.9054 | - | - | 1.14 [0.88, 1.40] | 1.26 [1.00, 1.53] |
| SDS | Assessment interview | 15.32 (7.98) | - | - | 7.71 (7.86) |  | 5.94 (7.45) |  | - | - | 0.96 [0.71, 1.21] | 1.21 [0.95, 1.47] |
| PHQ-9 | No guidance | 11.06 (6.17) | - | 6.82 (5.50) | 6.30 (5.08) | 0.4289 | 5.73 (5.00) | 0.1014 | - | 0.72 [0.48, 0.97] | 0.84 [0.59, 1.09] | 0.95 [0.70, 1.20] |
| PHQ-9 | Guidance | 10.42 (5.82) | - | 5.86 (4.51) | 5.40 (4.63) |  | 5.92 (5.25) |  | - | 0.87 [0.62, 1.13] | 0.95 [0.70, 1.21] | 0.81 [0.56, 1.06] |
| PHQ-9 | No assessment interview | 11.03 (6.22) | - | 6.02 (4.86) | 5.88 (4.91) | 0.7272 | 6.23 (5.58) | 0.4934 | - | 0.90 [0.64, 1.15] | 0.92 [0.66, 1.17] | 0.81 [0.56, 1.06] |
| PHQ-9 | Assessment interview | 10.45 (5.78) | - | 6.63 (5.24) | 5.78 (4.86) |  | 5.45 (4.61) |  | - | 0.69 [0.45, 0.93] | 0.87 [0.62, 1.12] | 0.95 [0.70, 1.20] |
| GAD-7 | No guidance | 8.68 (5.94) | - | - | 5.26 (4.35) | 0.4827 | 4.99 (4.10) | 0.9829 | - | - | 0.66 [0.41, 0.90] | 0.72 [0.48, 0.96] |
| GAD-7 | Guidance | 8.41 (5.93) | - | - | 4.69 (4.59) |  | 4.82 (5.14) |  | - | - | 0.70 [0.45, 0.95] | 0.64 [0.40, 0.89] |
| GAD-7 | No assessment interview | 8.68 (5.99) | - | - | 5.21 (4.53) | 0.5804 | 5.03 (4.87) | 0.8836 | - | - | 0.65 [0.41, 0.90] | 0.67 [0.42, 0.91] |
| GAD-7 | Assessment interview | 8.40 (5.87) | - | - | 4.74 (4.42) |  | 4.78 (4.41) |  | - | - | 0.70 [0.46, 0.95] | 0.69 [0.45, 0.94] |

Abbreviations: HDD= heavy drinking days, AUDIT= Alcohol Use Disorder Identification Test, PACS; Penn Alcohol Craving Scale BSCQ= Brief Situational Confidence Questionnaire, SDS = Sheehan Disability Scale, PHQ-9= Patient Health Questionnaire, GAD-7= Generalized Anxiety Disorder; ^*^ p<0.05
