# Supplementary material for: Optimizing internet-delivered cognitive behaviour therapy for alcohol misuse—a randomized factorial trial examining effects of a pre-treatment assessment interview and guidance
Source: Addict Sci Clin Pract. 2022 Jul 23;17:37. doi: 10.1186/s13722-022-00319-0 (PMC9308037; doi:10.1186/s13722-022-00319-0)
Supplement: Supplementary file 2 — Additional file 2: Table S2. Overall tests. [file 13722_2022_319_MOESM2_ESM.docx]

Additional file 2. Overall tests

|  |  | Test-statistic | p-value |
| --- | --- | --- | --- |
| TLFB | Assessment | 1.238 | 0.293 |
|  | Guidance | 0.846 | 0.496 |
| HDD | Assessment | 0.741 | 0.564 |
|  | Guidance | 0.613 | 0.653 |
| AUDIT | Assessment | 0.467 | 0.627 |
|  | Guidance | 0.304 | 0.737 |
| PACS | Assessment | 0.451 | 0.637 |
|  | Guidance | 0.078 | 0.925 |
| BSCQ | Assessment | 2.464 | 0.086 |
|  | Guidance | 1.525 | 0.219 |
| SDS | Assessment | 0.710 | 0.492 |
|  | Guidance | 2.459 | 0.087 |
| PHQ-9 | Assessment | 1.499 | 0.214 |
|  | Guidance | 1.350 | 0.257 |
| GAD-7 | Assessment | 0.147 | 0.863 |
|  | Guidance | 0.322 | 0.725 |
